# The mortality risk of night-time and daytime insomnia symptoms in an older population

**DOI:** 10.1038/s41598-023-36016-4

**Published:** 2023-06-13

**Authors:** Amy Harvey, Hannah Scott, Yohannes Adama Melaku, Leon Lack, Alexander Sweetman, Gorica Micic, Nicole Lovato

**Affiliations:** 1 College of Education, Psychology and Social Work, Flinders University, GPO Box 2100, Adelaide, 5001 Australia; 2 Flinders Health and Medical Research Institute: Sleep Health, College of Medicine and Public Health, Flinders University, Adelaide, 5001 Australia; 3 Cancer Epidemiology Division, Cancer Council Victoria, Melbourne, VIC Australia

**Keywords:** Psychology, Risk factors, Health care, Geriatrics

## Abstract

The current study examined the association between insomnia symptoms and all-cause mortality in older adults (≥ 65 years). Data was used from 1969 older adults [*M* = 78 years, *SD* = 6.7 years] who participated in the Australian Longitudinal Study of Ageing. Insomnia symptoms were defined by nocturnal symptoms (difficulty falling asleep, difficulty maintaining sleep, early morning awakenings) and daytime symptoms (concentration difficulties, effort, inability to get going). Frequency of symptoms were combined to calculate an insomnia symptom score ranging from 0 (no symptoms) to 24 (sever symptoms) and quintiles of the score were constructed to provide a range of symptom severity. Multivariable Cox models were conducted to assess associations between insomnia symptom severity and mortality risk. In the median follow up of 9.2 years, there were 17,403 person-years at risk and the mortality rate was 8-per 100 person-years. Insomnia symptom severity was associated with increased mortality in the most severe quintile (adjusted HR_Q1vsQ5_ = 1.26, 95%CI [1.03–1.53], *p* = .02). Subsequent analyses showed this association was driven by daytime symptoms (adjusted HR_Q1vsQ5_ = 1.66, [1.39–2.00], *p* < .0001), since nocturnal symptoms alone were not associated with increased mortality (adjusted HR _Q1vsQ5_ = 0.89, [0.72–1.10], *p* = .28). Findings suggest daytime symptoms drive increased mortality risk associated with insomnia symptoms. Findings may be therapeutically helpful by reassuring individuals with nocturnal insomnia symptoms alone that their longevity is unlikely to be impacted.

## Introduction

Insomnia disorder is estimated to affect approximately 770 million people worldwide, with an estimated 2.5 million individuals affected in Australia alone^1,2^. Insomnia disorder includes nocturnal symptoms of difficulties falling asleep, maintaining sleep and/or early morning awakenings with an inability to fall back to sleep^3^. An important criterion, and pertinent to this study, is that the sleep disturbance must also be accompanied by significant daytime impairment, such as memory or concentration difficulties, daytime fatigue, sleepiness, and/or negative mood^3,4^. In addition, to meet diagnostic criteria for the disorder^3^, these nocturnal and daytime symptoms must occur at least three times a week, for a duration of at least three months despite adequate opportunity to sleep.

Empirical evidence suggests that the symptoms of insomnia are strongly associated with significant medical comorbidity. Several studies have documented associations between insomnia symptoms and psychiatric disorders, namely depression and anxiety^5–8^, increased cardio-metabolic disease risk, including hypertension^9–11^, diabetes^12,13^, and inflammatory diseases^14^. Due to the increased prevalence of self-harm and suicide seen in psychiatric disorders (e.g., depression), the comorbidity of insomnia symptoms with these psychiatric disorders may indirectly increase the risk of mortality^1^. Furthermore, several studies have found individuals with insomnia symptoms experience increased activation of the hypothalamic–pituitary–adrenal (HPA^15^) axis and autonomic nervous system, which can cause increased heart rate and irregular heart rate variability^16–19^. This overactivation has been associated with hypertension, diabetes, and ultimately, increased mortality risk^1,20,21^.

Despite consistent associations between insomnia symptoms and morbidity, empirical evidence linking insomnia symptoms and mortality is unclear. Whilst some studies support a positive association between symptoms of insomnia and mortality risk^22–24^ others report no association^25–29^, or even a negative association^30^. In a sample of 3430 individuals aged 35 years or older, Chein and colleagues found a 15% increase in mortality risk for those experiencing frequent insomnia symptoms compared to those without symptoms^22^. Similarly, in a recent study of 15,511 individuals aged 50 years or older, Mahmood and colleges^31^ found significant associations between selected nocturnal insomnia symptoms (i.e., difficultly initiating sleep, early morning awakenings, nonrestorative sleep) and all-cause mortality. Contrastingly, in a sample of 1.1 million adults aged 30 years or older, Kripke and colleagues found a 14% reduction in mortality risk for those with insomnia symptoms compared to those who do not experience symptoms of insomnia^30^. A recent meta-analysis^1^ of 17-studies including over 36 million individuals found no difference in mortality risk for those with or without symptoms of insomnia after controlling for potential confounders^1^ (e.g., age, sex, marital status). This meta-analysis highlights uncertainties about mortality risk given methodological differences between individual studies.

One major limitation of previous studies is the absence of assessment for daytime symptoms of insomnia. This is despite daytime impairment being a core criterion for diagnosis^3,32^. Additionally, several studies investigating the relationship between the symptoms of insomnia and mortality risk have conducted short follow-up assessments after baseline measures (e.g., ≤ 6 years)^30,33–36^. The occurrence of death within short follow-up periods is highly unlikely, resulting in a smaller number of events (e.g., 6.4–30.5%)^30,33–36^. Furthermore, previous studies have dichotomised samples as either having or not having insomnia symptoms^22,24,27,29^. Potential drawbacks of dichotomising insomnia include; a reduction of statistical power, arbitrary decisions about the appropriate cut-point, loss of information about the effect of different levels of insomnia symptom severity on mortality, and masking of any non-linear relationships between insomnia symptoms and mortality^37^.

The present study assessed the association between insomnia symptom severity as a continuous variable and mortality risk. This enabled assessment of the full range of insomnia symptom severity and mortality risk, rather than dichotomising into insomnia symptoms versus no insomnia symptoms. A large, population-based cohort of older adults (≥ 65 years) was followed over a sufficiently long duration to result in a substantial mortality rate. In accordance with diagnostic classification systems, the present study assessed nocturnal and common daytime symptoms of insomnia in relation to mortality risk, both in combination and separately to shed light on previous findings. This is the first study to account for these previous methodological limitations when examining the link between insomnia symptoms and mortality risk.

## Method

### Participants

This study is an archival analysis of data from the 20-year long Australian Longitudinal Study of Ageing (ALSA)^38^. Participants consisted of adults aged 65 years or older who resided in the Adelaide Statistical Division on 31 December 1992^39^. Participants were randomly selected using the South Australian Electoral Roll, with the sample stratified by age group (i.e., 65–79, 70–74, 75–79, 80–84, and ≥ 85 years), sex, and local government area. Participants were community-dwelling adults and those living in residential care^39^. Letters of introduction and invitations to participate were sent to 3263 prospective participants^39^.

Of the 3263 people initially contacted, 506 were not eligible to participate (210 were deceased, 88 were not fluent in English language and no translator was available, 189 were not contactable, 37 were currently residing outside the Adelaide Statistical Division, and 36 for other reasons)^39^. Of the 2703 eligible participants, 1477 consented to participate^39^. To increase sample size, spouses and other household members were also invited to participate and original inclusion criterion of 70 years was relaxed to 65 years, resulting in an additional recruitment of 597 spouses and 13 household members^39^. In total, 2087 people participated at baseline (Wave 1)^39^. Informed consent was obtained from all participants.

### Materials

Baseline data was obtained through structured interviews conducted in-person at the participant’s usual place of residence^39^ between September 1992 and February 1993^39^. Participants were asked to respond to a set of closed-ended questions read by a graduate trained in standard assessment^39^. ALSA collated data from a wide range of domains, however only domains relevant to the present study will be discussed. Information regarding demographic characteristics (i.e., age, sex, marital status, education), non-communicable diseases (i.e., cancer, cardiovascular disease, depression, diabetes), sleep duration, and hypnotic medication use were obtained during interviews at baseline (Wave 1). All methods were carried out in accordance with relevant guidelines and regulations^38^. Additionally, all experimental protocols were approved by the Committee on Clinical Investigation at Flinders Medical Centre in 1988 and were subject to regular ethical review through the active study period^38^.

### Measures

#### Insomnia symptoms

Nocturnal symptoms of insomnia were assessed using three items: *“How often do you have trouble falling asleep”*, *“How often do you have trouble with waking up during the night?”* and *“How often do you have trouble with waking up earlier than intended and not being able to fall asleep again at all?”*. Participants were asked to respond to each item on a five-point Likert scale (0 = *never* to 4 = *almost always*). Scores from each item were summed to produce a total nocturnal symptom score (0 = *no nocturnal symptoms* to 12 = *most severe nocturnal symptoms*).

Daytime symptoms of insomnia were assessed using four items*: “I was bothered by things that usually don’t bother me”*, *“I had trouble keeping my mind on what I was doing”*, *“I felt like everything I did was an effort”* and *“I could not get going”*. Participants reported the frequency of each item in the past week on a four-point Likert scale (0 = *rarely or none of the time* to 3 = *most or all of the time*). Scores from each item were summed to produce a total daytime symptom score (0 = *no daytime symptoms* to 12 = *most severe daytime symptoms).* A principle component factor analysis was conducted on CES-D items to identify the best items for use in assessing daytime symptoms of insomnia disorder. Using varimax rotation, four factors had eigenvalues greater than 1. However, subsequent examination of the items loading on to the four factors revealed that three factors were more conceptually interpretable: affect, somatic/effort, and perception of other’s opinions. All items chosen as daytime symptoms for insomnia in the present study (i.e., bother, concentrate, effort, get going) loaded highly onto somatic/effort (factor 2), along with the sleep-related item of the CES-D (i.e., my sleep was restless), and had low correlations with the affect-related symptoms of depression (factor 1). These findings suggest items chosen to represent daytime symptoms of insomnia disorder in the present study do not represent the melancholic symptoms of depression, but instead may better represent daytime symptoms of insomnia disorder.

Total insomnia symptom severity scores were calculated by summing nocturnal and daytime symptom total scores (0 = *no insomnia symptoms*, 24 = *most severe insomnia symptoms*).

#### Mortality

The dependent variable was the occurrence of death between the baseline interviews conducted between 1992 and 1993^39^ and 14.3 years later in 2006. Date of death was assessed via the Adelaide Registry of Births, Deaths and Marriages^39^. In cases where participants died outside of the Adelaide Statistical Division or died between registry reports, relatives and other informants supplied date of death^39^.

#### Covariates

Demographic characteristics including sex, age, marital status, and education were controlled for in adjusted models due to established associations with insomnia and mortality risk^2,40–43^. Marital status was reported in six categories (*married*, de facto, *separated*, *divorced*, *widowed*, *never married*). Tertiary education was reported dichotomously (*yes*, *no*). Non-communicable diseases reported at baseline, including cancer^44^, cardiovascular disease (CVD)^45^, depressive symptoms^2,5,6,8^ and diabetes^2,12,13^ were controlled for in adjusted models due to established associations with mortality risk. Depressive symptoms were measured using the CES-D with the sleep item removed, with scores from each item summed to create a continuous measure^46^. Scores ranged from 0 to 60, with higher scores indicating more severe depressive symptoms^46^. The recommended cut-off score of ≥ 16 was adopted to reflect symptoms of depression^46^. Sleep duration was controlled for in adjusted models due to a known u-shaped relationship between sleep duration, insomnia and mortality^24,47,48^. Hypnotic medication use was also controlled for due to its associations with insomnia and mortality risk^30,33^.

### Statistical analysis

Kaplan–Meier Survival Estimates^49^ were used to compare survival probability of participant by severity of insomnia symptoms. Cox models were used to estimate hazard ratios and 95% confidence intervals on the association between insomnia symptoms and mortality. The present study controlled for the confounding effects of demographic characteristics (age, sex, marital status and education), non-communicable diseases (cancer, cardiovascular disease, depression, diabetes), sleep duration, and hypnotic use. Insomnia symptom severity scores were divided into quintiles (i.e., 20% of the total sample within each quintile). Quintile 1 (reference) represented the bottom fifth of symptom scores (i.e., 20% of the population with the lowest symptom severity scores), and Quintile 5 represented the top fifth severity scores (i.e., 20% of the population with the highest symptom severity scores). The associations between nocturnal and daytime symptoms separately with mortality were also examined to further elucidate the association between insomnia symptoms and mortality. A correlation between nocturnal and daytime symptoms was also conducted to determine the strength of the association between these insomnia symptoms. Stata version 16^50^ was used to conduct all analyses.

Sensitivity analyses were conducted in addition to the main analyses described above. The association between insomnia symptoms (as a combined score and separately for nocturnal and daytime symptoms) and mortality were explored after excluding participants with depression and after excluding participants with chronic diseases. The findings of these analyses are provided in the Supplementary Materials and are consistent with the findings presented below.

### Statement of significance

Prior research has found mixed findings on the association between symptoms of insomnia and mortality risk. The current study potentially elucidated these inconsistencies by examining separately the daytime and night-time symptoms of insomnia and their relation to mortality risk in a large longitudinal study of Australian older adults. The findings indicate that increased mortality risk associated with insomnia symptoms may be entirely driven by the presence of daytime symptoms, with no association found between night-time symptoms and mortality risk. This suggests that daytime impairment, which may or may not be directly attributable to symptoms of insomnia, increases mortality risk. Additionally, the presence of night-time symptoms without daytime symptoms may not be detrimental, at least from the perspective of mortality risk.

## Results

### Participant characteristics

Participants’ mean age at baseline was 78 years (*SD* = 6.7 years), with a similar proportion of male and female participants (51% and 49%, respectively). After excluding missing data (*n* = 118), 1,969 participants were included in this study. Data from 118 participants were removed due to missing values on measures of sleep duration (*n* = 92), insomnia score (*n* = 48), qualification (*n* = 20), night-time symptom score (*n* = *18*)*,* cancer diagnosis (*n* = 7), diabetes (*n* = 1), and marital status (*n* = 1)*.* A total of 1377 participants (69.9%) had died by a median follow-up time of 9.2 years. Baseline demographic characteristics, non-communicable diseases, sleep duration and hypnotic use are listed in Table 1, according to insomnia symptom frequency quintiles. The quintile thresholds were round numbers and as such, quintiles are not precisely 20% of the study sample. Compared to those in Quintile 1 (i.e., those experiencing little to no insomnia symptoms), participants in Quintile 5 (i.e., those experiencing severe insomnia symptoms) were more likely to be older, female, and regularly use hypnotic medications. They were also more likely to have depressive symptoms, a history of CVD, a typical sleep duration of < 6 h, and were less likely to have a history of cancer, and tertiary education.

**Table 1:** Baseline demographic, non-communicable diseases, sleep duration and hypnotic use according to insomnia severity quintiles.

Characteristics	Quintiles					Total Sample	*P*-Value
	1	2	3	4	5		
Quintile Cut-Offs	3	4	7	9			
N, %	575, 29.2	218, 11.1	536, 27.2	255, 13	385, 19.6	1969, 100	
Age*, y (SD)	77.6 (6.5)	77.9 (7.2)	77.4 (6.4)	78.4 (6.8)	78.8 (6.7)		.014
Sex˚							< .0001
Male, %	336, 58.4	120, 55	272, 50.7	125, 49	151, 39.2	1004, 51	
Female, %	239, 41.6	98, 45	264, 49.3	130, 51	234, 60.8	965, 49	
Marital Status˚							.077
Married, %	399, 69.4	142, 65.1	358, 66.8	168, 65.9	234, 60.8	1301, 66.1	
De Facto, %	2, 0.3	0, 0	3, 0.6	0, 0	0, 0	5, 0.3	
Separated, %	2, 0.3	3, 1.4	5, 0.9	4, 1.6	1, 0.3	15, 0.8	
Divorced, %	10, 1.7	1, 0.5	11, 2.1	3, 1.2	8, 2.1	33, 1.7	
Widowed, %	144, 25	62, 28.4	139, 25.9	70, 27.5	129, 33.5	544, 27.6	
Never Married, %	18, 3.1	10, 4.6	20, 3.7	10, 3.9	13, 3.4	71, 3.6	
Tertiary Education˚, %	217, 37.7	85, 39	193, 36	79, 31	106, 27.5	680, 34.5	.005

Characteristics	Quintiles					Total Sample	*P*-Value
	1	2	3	4	5		
History of cancer°, %	64, 11.1	24, 16.2	87, 16.2	48, 18.8	54, 14	277, 14.1	.013
History of CVD°, %	93, 16.2	24, 11	103, 19.2	47, 18.4	107, 27.8	374, 19	< .0001
Depression°, %	8, 1.4	7, 3.2	48, 9	33, 12.9	185, 48.1	281, 14.3	< .0001
History of diabetes°, %	46, 8	20, 9.2	46, 8.6	20, 7.8	40, 10.4	172, 8.7	.730
Sleep duration°							< .0001
< 6 h, %	39, 6.8	16, 7.3	99, 18.5	94, 36.9	196, 50.9	444, 22.5	
6–8.99 h, %	431, 75	163, 74.8	385, 71.8	149, 58.4	168, 43.6	1296, 65.8	
≥ 9 h, %	105, 18.3	39, 17.9	52, 9.7	12, 4.7	21, 5.5	229, 11.6	
Hypnotic Use°							< .0001
Nightly, %	44, 7.7	31, 14.2	62, 11.6	41, 16.1	106, 27.5	284, 14.4	
Few times per week, %	10, 1.7	4, 1.8	19, 3.5	13, 5.1	38, 9.9	84, 4.3	
Few times per month, %	12, 2.1	5, 2.3	27, 5	12, 4.7	33, 8.6	89, 4.5	
Less often, %	25, 4.3	14, 6.4	54, 10.1	20, 7.8	36, 9.4	149, 7.6	
Never, %	484, 84.2	163, 74.8	373, 69.6	169, 66.3	172, 44.7	1361, 69.2	

Data presented as total within quintile, % of sample within quintile.

### Insomnia symptoms and mortality risk

**Figure 1:**
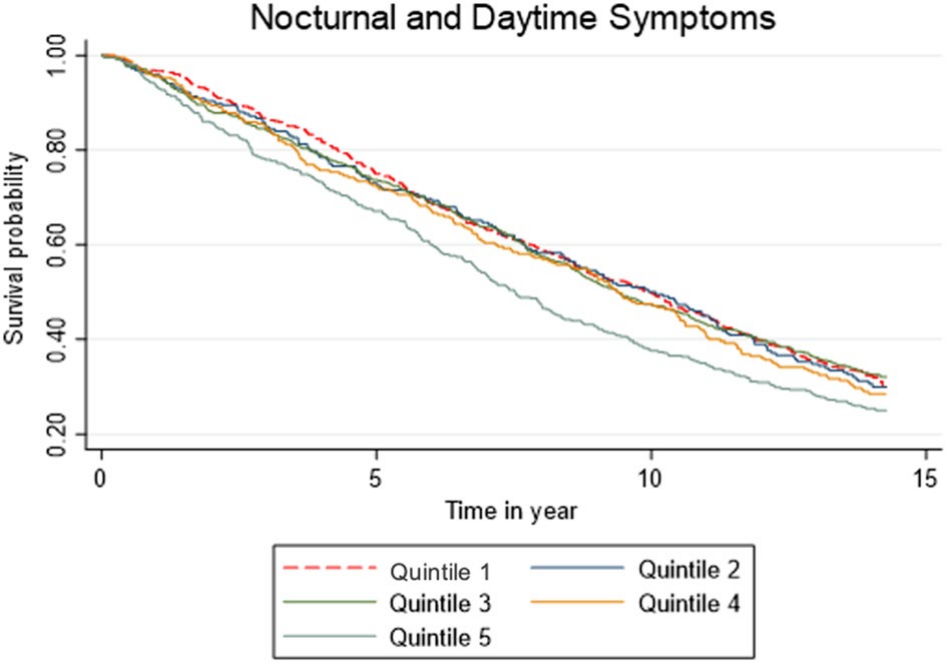
Kaplan–Meier curve for insomnia symptom severity in an unadjusted analysis.

As seen in Fig. 1, Kaplan–Meier curve showed insomnia symptom severity was significantly associated with mortality risk (*p* = 0.003). Participants in Quintile 5 had significantly increased mortality risk compared to Quintile 1 (HR = 1.28, 95%CI [1.10–1.49], *p* = 0.001). However, participants in Quintiles 2 (HR = 1.03, [0.86–1.24], *p* = 0.74), 3 (HR = 1.01, [0.87–1.16], *p* = 0.94), and 4 (HR = 1.09, [0.91–1.30], *p* = 0.33) had no increased mortality risk compared to Quintile 1.

After adjustment for demographic characteristics (i.e., age, sex, marital status, education), non-communicable diseases (i.e., cancer, depression, CVD, diabetes), sleep duration, and hypnotic use, insomnia symptom severity was significantly associated with mortality risk (*p* = 0.01). As seen in Table 2 (with HRs for each model step shown in the Supplementary File), participants in Quintile 5 still had significantly increased mortality risk in the adjusted model compared to Quintile 1 (HR = 1.26, 95% CI [1.03–1.53], *p* = 0.02). No associations between insomnia symptom severity and mortality risk were observed for participants in Quintiles 2 (HR = 1.04, [0.86–1.26], *p* = 0.65), 3 (HR = 1.14, [0.99–1.32], *p* = 0.07), and 4 (HR = 1.17, [0.97–1.40], *p* = 0.1) compared to Quintile 1 in the adjusted model.

**Table 2:** Insomnia symptom severity in a fully adjusted analysis.

Quintiles	HR	SE	z	*P* > z	95% lower CI	95% upper CI	*P* for trend
							0.0130
Q1	–	–	–	–	–	–	
Q2	1.04	0.10	0.45	0.653	0.86	1.26	
Q3	1.14	0.09	1.77	0.076	0.99	1.32	
Q4	1.17	0.11	1.63	0.104	0.97	1.40	
Q5	1.26	1.13	2.27	0.023	1.03	1.53	

*Adjusted for age, sex, marital status, education, communicable diseases (cancer, cardiovascular disease, depression, diabetes), sleep duration, and hypnotic use.

**Figure 2:**
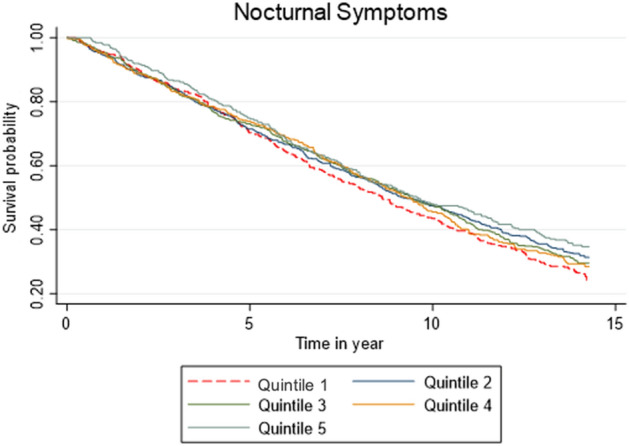
Kaplan–Meier survival estimates for nocturnal symptom frequency in a fully adjusted analysis.

To elucidate possible mechanisms for the significant association between insomnia symptom severity and mortality risk, nocturnal and daytime symptoms were considered separately. Using the adjusted model, a Cox regression model showed the association between the combined nocturnal symptoms score and mortality risk were not significant (*p* = 0.87). Estimates, seen in Fig. 2, showed the association between nocturnal symptom severity and mortality risk were not significant for all quintiles. Table 3 shows the hazard ratios and 95% CI for each quintile in the fully adjusted were as follows: Quintile 2 (HR = 0.89, 95% CI [0.76–1.04], *p* = 0.13), Quintile 3 (HR = 0.93, [0.79–1.09], *p* = 0.36), Quintile 4 (HR = 0.96, [0.80–1.15], *p* = 0.67), and Quintile 5 (HR = 0.89, [0.72–1.10], *p* = 0.28). See the Supplementary File for HRs at each model step.

**Table 3:** Nocturnal symptom severity in a fully adjusted analysis.

Quintiles	HR	SE	z	*P* > z	95% lower CI	95% upper CI	P for trend
							0.509
Q1	–	–	–	–	–	–	
Q2	0.89	0.07	− 1.49	0.137	0.76	1.04	
Q3	0.93	0.08	− 0.91	0.363	0.79	1.09	
Q4	0.96	0.09	− 0.43	0.665	0.80	1.15	
Q5	0.89	0.10	− 1.09	0.276	0.72	1.10	

*Adjusted for age, sex, marital status, education, communicable diseases (cancer, cardiovascular disease, depression, diabetes), sleep duration, and hypnotic use.

**Figure 3:**
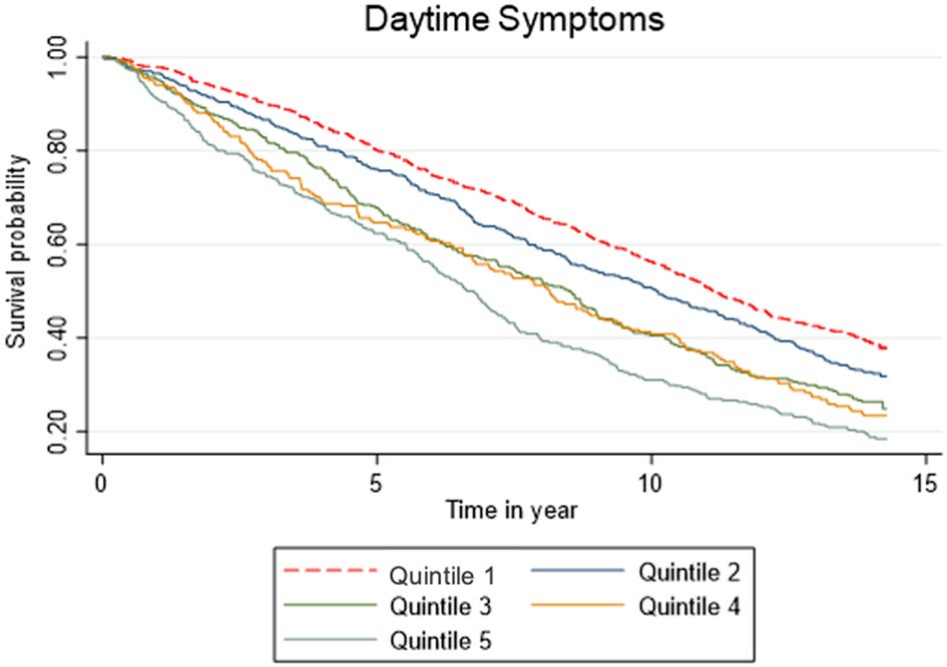
Kaplan–Meier survival estimates for daytime symptom frequency in a fully adjusted analysis.

Estimates, seen in Fig. 3*,* showed the association between the combined daytime symptoms score and mortality risk was significant (*p* < 0.0001). As seen in Table 4 (with HRs for each model step shown in the Supplementary File)*,* participants in Quintile 5 had significantly increased mortality risk compared to Quintile 1 in the adjusted model (Fig. 3; HR = 1.66, 95% CI [1.39–2.00], *p* < 0.0001). Additionally, participants in Quintiles 2 (HR = 1.20, [1.03–1.40], *p* = 0.02), 3 (HR = 1.45, [1.23–1.71], *p* < 0.0001), and 4 (HR = 1.35, [1.12–1.63], *p* < 0.0001) also had significantly increased mortality risk compared to Quintile 1.

**Table 4:** Daytime symptom severity in a fully adjusted analysis.

Quintiles	HR	SE	z	*P* > z	95% lower CI	95% upper CI	*P* for trend
							< 0.0001
Q1	–	–	–	–	–	–	
Q2	1.20	0.09	2.38	0.017	1.03	1.40	
Q3	1.45	0.12	4.40	< 0.0001	1.23	1.71	
Q4	1.35	0.13	3.11	0.002	1.12	1.63	
Q5	1.66	0.16	5.46	< 0.0001	1.39	2.00	

*Adjusted for age, sex, marital status, education, communicable diseases (cancer, cardiovascular disease, depression, diabetes), sleep duration, and hypnotic use.

A correlation was conducted between nocturnal and daytime symptoms to help elucidate whether the daytime symptoms co-occurred with (and were therefore potentially caused by) the nocturnal symptoms. There was a small, significant correlation between nocturnal and daytime symptoms, r_(s)_ = 0.21, *p* < 0.001.

## Discussion

The present study aimed to determine whether insomnia symptoms were associated with an increased mortality risk. Results showed that insomnia symptom severity (combining nocturnal and daytime symptoms) was significantly associated with mortality risk in the adjusted analysis, with the top quintile of insomnia symptom severity having a significantly elevated mortality risk compared to the lowest quintile. Subsequent analyses found that this association appeared to be entirely driven by daytime symptoms, since nocturnal symptoms were not associated with elevated mortality risk.

Findings from the present study support some previous literature on the association between symptoms of insomnia and mortality. Many studies that only accounted for nocturnal symptoms in their definition of insomnia found no association with mortality risk (as found in this study), or even a reduced association with mortality^25,26,28–30^. In contrast, studies that accounted for both nocturnal and daytime symptoms of insomnia largely found an association between insomnia symptoms and mortality^1,23,51^. The current study suggests that this prior relationship may be entirely driven by the degree of daytime symptoms. Accordingly, future research should consider the presence of both nocturnal and daytime symptoms of insomnia when evaluating health outcomes, as the presence/absence of daytime symptoms appears to greatly impact findings.

While insomnia requires both nocturnal and daytime symptoms for a diagnosis, the lack of any relationship between nocturnal symptoms and mortality risk suggests that the increased mortality risk may not be associated with the disorder. As we would expect that the two symptom types would be highly correlated if the daytime symptoms were caused by the insomnia, we would expect nocturnal symptoms to also be associated with mortality risk^52^. Since no association was found between nocturnal symptoms and mortality risk, this suggests that daytime symptoms that were associated with the increased mortality risk were likely caused by a condition other than insomnia. One explanation for the association of daytime symptoms with mortality risk may be that these symptoms are indicative of health conditions not assessed within the context of this study, or undiagnosed health conditions present at baseline. Such covert health conditions could not be controlled in the statistical analysis. For example, Garland and colleagues theorised that daytime symptoms of fatigue may represent early warning signs of cancer, which in turn increases mortality risk^53^. As fatigue is also a common daytime complaint of insomnia disorder, the presence of fatigue at baseline could have been feasibly due to insomnia symptoms or to another condition like cancer. This notion is reinforced by the small correlation between nocturnal and daytime symptoms (r = 0.21, *p* < 0.001), whereby about 4% of the variance of the daytime symptoms is accounted for by the nocturnal symptoms. This indicates these symptoms are largely independent and due to a large array of other contributing factors (e.g. physical fitness, physical health, covert morbidities, daily stresses, mental health). Future research should further elucidate the attribution of daytime symptoms to health conditions to further examine the relationship between insomnia symptoms and mortality risk.

These findings may hold significant therapeutic weight. A commonly-held belief by those with insomnia disorder is that their disorder is detrimental to health, which is particularly observed amongst older adults with comorbid medical conditions^1^. As no association was found between nocturnal symptoms and mortality risk, even for those who frequently experience nocturnal symptoms, incorporating these findings into treatments such as Cognitive Behavioral Therapy for Insomnia (CBTi^54^) may help to reduce anxiety and facilitate cognitive treatment efficacy. In addition, the present findings suggest that any residual daytime symptoms seen after treatment with CBTi must be assessed as they may indicate the presence of underlying health conditions, which could be associated with increased mortality risk.

The present study has several limitations that should be noted. Firstly, the present study did not control for the chronicity of insomnia symptoms. To meet diagnostic criteria for insomnia, symptoms must have occurred for at least three times a week for at least three months^3^. Although the present study did not directly control for chronicity, individuals with chronic insomnia, particularly those with more severe symptoms, do not typically remit within a 12-month period. This was supported by secondary analyses that found 85% of participants reported experiencing no changes to their sleep patterns within the last year^55^. Secondly, the reproducibility of the measurement tools used to assess nocturnal and daytime symptoms of insomnia is unknown, warranting further investigation to evaluate their reliability and consistency. Thirdly, as this is a correlational study, causation cannot be established: a common limitation of mortality risk research. Fourthly, the present study did not adjust for other sleep disorders, such as sleep apnea, which may be associated with both nocturnal and daytime symptoms of insomnia. The presence of these sleep disorders may potentially have influenced the reported associations between insomnia symptoms and mortality risk. Lastly, the questions used to assess daytime symptoms were not prefaced as the result of poor sleep, as is traditionally done in the assessment of insomnia (e.g., Insomnia Severity Index, Sleep Condition Indicator). This may, to some degree, account for a smaller correlation between nocturnal and daytime symptoms than would be expected. The measure of insomnia symptoms on a continuous scale rather than the presence/absence of insomnia disorder also limits the ability to compare these study findings to past research; however, the benefits of assessing the degree of insomnia symptoms may outweigh this disadvantage. Given these limitations, findings from the present study should be interpreted with some degree of caution.

The present study has raised many questions to address in future research. Firstly, to understand the temporal development of daytime symptoms and other medical/psychiatric conditions, future studies may assess people who are healthy at baseline and investigate how nocturnal/daytime insomnia symptoms and other diseases develop over time. Depending on the frequency of follow-up periods, this could test whether daytime symptoms lead to increased health conditions that cause increase mortality, or whether underlying health conditions lead to daytime symptoms that cause increased mortality. Future studies should also include cause of death data to gain a deeper understanding of underlying factors that may contribute to mortality risk. This data may enable researchers to develop more targeted interventions and improve clinical practices, leading to more effective strategies for reducing mortality risk in individuals experiencing symptoms of insomnia. Secondly, insomnia treatment studies with long-term follow-up periods would help to elucidate the potential mechanisms. For example, patients who alleviate daytime symptoms after treatment would be expected to have reduced mortality risk compared to those who do not, or those experience relapse sometime after treatment cessation. If this is found, it would suggest the association between daytime symptoms and mortality is caused by the insomnia. Thirdly, whilst the present study adjusted for hypnotic use, it is recommended that future studies conduct a stratified analysis that considers the use of hypnotic medications when examining the relationship between insomnia symptoms and all-cause mortality risk. This approach may provide further insights into how hypnotic use impacts the association between insomnia symptoms and mortality risk.

## Conclusion

The present study assessed whether insomnia symptoms were associated with mortality risk. Whilst initial analyses appeared to suggest insomnia symptoms were associated with increased risk of mortality, subsequent analyses showed this association was solely driven by daytime symptoms likely not caused by insomnia. These findings suggest that the symptoms of insomnia are not directly associated with mortality, although future research is needed to address limitations of this correlational study design. Findings from the present study hold significant implications for the treatment of insomnia symptoms. In closing, if the one question keeping you up at night is “will insomnia symptoms kill me?”, you may find reassurance in the present study findings.

## Supplementary Information

Supplementary Tables.

## Data Availability

The datasets analysed during the current study are held at the Flinders Centre for Ageing Studies (FCAS) at Flinders University of South Australia and are available to researchers upon reasonable request. Procedures for data access can be found on the Australian Longitudinal Study of Ageing website at https://sites.flinders.edu.au/alsa/.
