# Supplementary material for: The mortality risk of night-time and daytime insomnia symptoms in an older population
Source: Sci Rep. 2023 Jun 13;13:9575. doi: 10.1038/s41598-023-36016-4 (PMC10264459; doi:10.1038/s41598-023-36016-4)
Supplement: Supplementary file 1 — Supplementary Tables. [file 41598_2023_36016_MOESM1_ESM.docx]

**Supplementary Materials**

Hazard ratios for mortality by insomnia symptom quintile, for each model step:

***Insomnia symptoms combined***

| **Model 1 – Adjusted for age, sex, marital status, qualifications** | | | | | | | |
| --- | --- | --- | --- | --- | --- | --- | --- |
| **Quintiles** | **HR** | **SE** | **z** | **P>z** | **95% lower CI** | **95% upper CI** | **P for trend** |
|  |  |  |  |  |  |  | 0.0030 |
| Q1 | - | - | - | - | - | - |  |
| Q2 | 1.03 | 0.10 | 0.33 | 0.742 | 0.86 | 1.24 |  |
| Q3 | 1.01 | 0.07 | 0.08 | 0.939 | 0.87 | 1.16 |  |
| Q4 | 1.09 | 0.10 | 0.97 | 0.333 | 0.91 | 1.30 |  |
| Q5 | 1.28 | 0.10 | 3.18 | 0.001 | 1.10 | 1.49 |  |

| **Model 2 – Model 1 + cancer, CVD, depression, diabetes, total sleep time** | | | | | | | |
| --- | --- | --- | --- | --- | --- | --- | --- |
| **Quintiles** | **HR** | **SE** | **z** | **P>z** | **95% lower CI** | **95% upper CI** | **P for trend** |
|  |  |  |  |  |  |  | 0.0020 |
| Q1 | - | - | - | - | - | - |  |
| Q2 | 1.05 | 0.10 | 0.52 | 0.600 | 0.87 | 1.27 |  |
| Q3 | 1.16 | 0.09 | 2.01 | 0.045 | 1.00 | 1.34 |  |
| Q4 | 1.20 | 0.11 | 1.91 | 0.056 | 1.00 | 1.44 |  |
| Q5 | 1.33 | 0.13 | 2.88 | 0.004 | 1.10 | 1.61 |  |

| **Model 3 – Model 2 + sedative use** | | | | | | | |
| --- | --- | --- | --- | --- | --- | --- | --- |
| **Quintiles** | **HR** | **SE** | **z** | **P>z** | **95% lower CI** | **95% upper CI** | **P for trend** |
|  |  |  |  |  |  |  | 0.0130 |
| Q1 | - | - | - | - | - | - |  |
| Q2 | 1.04 | 0.10 | 0.45 | 0.653 | 0.86 | 1.26 |  |
| Q3 | 1.14 | 0.09 | 1.77 | 0.076 | 0.99 | 1.32 |  |
| Q4 | 1.17 | 0.11 | 1.63 | 0.104 | 0.97 | 1.40 |  |
| Q5 | 1.26 | 1.13 | 2.27 | 0.023 | 1.03 | 1.53 |  |

***Night-time symptoms***

| **Model 1 – Adjusted for age, sex, marital status, qualifications** | | | | | | | |
| --- | --- | --- | --- | --- | --- | --- | --- |
| **Quintiles** | **HR** | **SE** | **z** | **P>z** | **95% lower CI** | **95% upper CI** | **P for trend** |
|  |  |  |  |  |  |  | 0.064 |
| Q1 | - | - | - | - | - | - |  |
| Q2 | 0.88 | 0.07 | -1.63 | 0.102 | 0.75 | 1.03 |  |
| Q3 | 0.91 | 0.07 | -1.24 | 0.214 | 0.78 | 1.06 |  |
| Q4 | 0.92 | 0.08 | -0.91 | 0.361 | 0.78 | 1.10 |  |
| Q5 | 0.81 | 0.07 | -2.37 | 0.018 | 0.68 | 0.96 |  |

| **Model 2 – Model 1 + cancer, CVD, depression, diabetes, total sleep time** | | | | | | | |
| --- | --- | --- | --- | --- | --- | --- | --- |
| **Quintiles** | **HR** | **SE** | **z** | **P>z** | **95% lower CI** | **95% upper CI** | **P for trend** |
|  |  |  |  |  |  |  | 0.868 |
| Q1 | - | - | - | - | - | - |  |
| Q2 | 0.89 | 0.07 | -1.43 | 0.152 | 0.76 | 1.04 |  |
| Q3 | 0.94 | 0.08 | -0.72 | 0.471 | 0.80 | 1.11 |  |
| Q4 | 0.99 | 0.09 | -0.15 | 0.880 | 0.82 | 1.18 |  |
| Q5 | 0.94 | 0.10 | -0.60 | 0.551 | 0.76 | 1.16 |  |

| **Model 3 – Model 2 + sedative use** | | | | | | | |
| --- | --- | --- | --- | --- | --- | --- | --- |
| **Quintiles** | **HR** | **SE** | **z** | **P>z** | **95% lower CI** | **95% upper CI** | **P for trend** |
|  |  |  |  |  |  |  | 0.509 |
| Q1 | - | - | - | - | - | - |  |
| Q2 | 0.89 | 0.07 | -1.49 | 0.137 | 0.76 | 1.04 |  |
| Q3 | 0.93 | 0.08 | -0.91 | 0.363 | 0.79 | 1.09 |  |
| Q4 | 0.96 | 0.09 | -0.43 | 0.665 | 0.80 | 1.15 |  |
| Q5 | 0.89 | 0.10 | -1.09 | 0.276 | 0.72 | 1.10 |  |

***Daytime symptoms***

| **Model 1 – Adjusted for age, sex, marital status, qualifications** | | | | | | | |
| --- | --- | --- | --- | --- | --- | --- | --- |
| **Quintiles** | **HR** | **SE** | **z** | **P>z** | **95% lower CI** | **95% upper CI** | **P for trend** |
|  |  |  |  |  |  |  | <0.0001 |
| Q1 | - | - | - | - | - | - |  |
| Q2 | 1.19 | 0.09 | 2.29 | 0.022 | 1.03 | 1.39 |  |
| Q3 | 1.48 | 0.12 | 4.79 | <0.0001 | 1.26 | 1.74 |  |
| Q4 | 1.57 | 0.15 | 4.82 | <0.0001 | 1.31 | 1.89 |  |
| Q5 | 1.90 | 0.14 | 8.45 | <0.0001 | 1.64 | 2.20 |  |

| **Model 2 – Model 1 + cancer, CVD, depression, diabetes, total sleep time** | | | | | | | |
| --- | --- | --- | --- | --- | --- | --- | --- |
| **Quintiles** | **HR** | **SE** | **z** | **P>z** | **95% lower CI** | **95% upper CI** | **P for trend** |
|  |  |  |  |  |  |  | <0.0001 |
| Q1 | - | - | - | - | - | - |  |
| Q2 | 1.21 | 0.09 | 2.50 | 0.012 | 1.04 | 1.41 |  |
| Q3 | 1.47 | 0.12 | 4.59 | <0.0001 | 1.25 | 1.73 |  |
| Q4 | 1.38 | 0.13 | 3.34 | 0.0010 | 1.14 | 1.66 |  |
| Q5 | 1.71 | 0.16 | 5.82 | <0.0001 | 1.43 | 2.05 |  |

| **Model 3 – Model 2 + sedative use** | | | | | | | |
| --- | --- | --- | --- | --- | --- | --- | --- |
| **Quintiles** | **HR** | **SE** | **z** | **P>z** | **95% lower CI** | **95% upper CI** | **P for trend** |
|  |  |  |  |  |  |  | <0.0001 |
| Q1 | - | - | - | - | - | - |  |
| Q2 | 1.20 | 0.09 | 2.38 | 0.017 | 1.03 | 1.40 |  |
| Q3 | 1.45 | 0.12 | 4.40 | <0.0001 | 1.23 | 1.71 |  |
| Q4 | 1.35 | 0.13 | 3.11 | 0.002 | 1.12 | 1.63 |  |
| Q5 | 1.66 | 0.16 | 5.46 | <0.0001 | 1.39 | 2.00 |  |

Associations between insomnia symptoms and mortality, excluding participants with depression:

| **Insomnia symptoms combined** | **HR** | **SE** | **z** | **P>z** | **95% lower CI** | **95% upper CI** | **P for trend** | |
| --- | --- | --- | --- | --- | --- | --- | --- | --- |
|  | | | | | | | | <0.0001 |
| Q1 | - | - | - | - | - | - |  | |
| Q2 | 1.05 | 0.10 | 0.49 | 0.625 | 0.87 | 1.27 |  | |
| Q3 | 1.16 | 0.09 | 1.97 | 0.049 | 1.00 | 1.34 |  | |
| Q4 | 1.20 | 0.11 | 1.93 | 0.053 | 1.00 | 1.44 |  | |
| Q5 | 1.42 | 0.13 | 3.95 | 0.000 | 1.19 | 1.69 |  | |

| **Nocturnal Symptoms** | **HR** | **SE** | **z** | **P>z** | **95% lower CI** | **95% upper CI** | **P for trend** | |
| --- | --- | --- | --- | --- | --- | --- | --- | --- |
|  | | | | | | | | 0.984 |
| Q1 | - | - | - | - | - | - |  | |
| Q2 | 0.90 | 0.07 | -1.27 | 0.205 | 0.77 | 1.06 |  | |
| Q3 | 0.94 | 0.08 | -0.73 | 0.463 | 0.80 | 1.10 |  | |
| Q4 | 1.02 | 0.09 | 0.17 | 0.861 | 0.85 | 1.22 |  | |
| Q5 | 0.95 | 0.10 | -0.48 | 0.632 | 0.77 | 1.17 |  | |

| **Daytime Symptoms** | **HR** | **SE** | **z** | **P>z** | **95% lower CI** | **95% upper CI** | **P for trend** | |
| --- | --- | --- | --- | --- | --- | --- | --- | --- |
|  | | | | | | | | <0.0001 |
| Q1 | - | - | - | - | - | - |  | |
| Q2 | 1.204 | 0.094 | 2.39 | 0.017 | 1.034 | 1.403 |  | |
| Q3 | 1.452 | 0.122 | 4.45 | 0 | 1.232 | 1.712 |  | |
| Q4 | 1.360 | 0.130 | 3.22 | 0.001 | 1.128 | 1.640 |  | |
| Q5 | 1.734 | 0.137 | 6.97 | 0 | 1.485 | 2.024 |  | |

Note: All analyses adjusted for age, sex, marital status, qualifications, cancer, cardiovascular disease, diabetes, total sleep time, sedative use.

Associations between insomnia symptoms and mortality, excluding participants with chronic disease:

| **Insomnia symptoms combined** | **HR** | **SE** | **z** | **P>z** | **95% lower CI** | **95% upper CI** | **P for trend** | |
| --- | --- | --- | --- | --- | --- | --- | --- | --- |
|  | | | | | | | | 0.066 |
| Q1 | - | - | - | - | - | - |  | |
| Q2 | 1.11 | 0.12 | 0.95 | 0.341 | 0.90 | 1.37 |  | |
| Q3 | 1.16 | 0.14 | 1.23 | 0.217 | 0.92 | 1.48 |  | |
| Q4 | 1.21 | 0.16 | 1.44 | 0.151 | 0.93 | 1.57 |  | |
| Q5 | 1.27 | 0.17 | 1.72 | 0.085 | 0.97 | 1.66 |  | |

| **Nocturnal Symptoms** | **HR** | **SE** | **z** | **P>z** | **95% lower CI** | **95% upper CI** | **P for trend** | |
| --- | --- | --- | --- | --- | --- | --- | --- | --- |
|  | | | | | | | | 0.857 |
| Q1 | - | - | - | - | - | - |  | |
| Q2 | 0.921 | 0.101 | -0.75 | 0.452 | 0.744 | 1.141 |  | |
| Q3 | 0.961 | 0.123 | -0.31 | 0.755 | 0.748 | 1.234 |  | |
| Q4 | 0.991 | 0.121 | -0.07 | 0.942 | 0.780 | 1.260 |  | |
| Q5 | 1.004 | 0.139 | 0.03 | 0.976 | 0.765 | 1.318 |  | |

| **Daytime Symptoms** | **HR** | **SE** | **z** | **P>z** | **95% lower CI** | **95% upper CI** | **P for trend** | |
| --- | --- | --- | --- | --- | --- | --- | --- | --- |
|  | | | | | | | | <0.0001 |
| Q1 and Q2 | - | - | - | - | - | - |  | |
| Q3 | 1.253 | 0.125 | 2.26 | 0.024 | 1.031 | 1.524 |  | |
| Q4 | 1.439 | 0.160 | 3.28 | 0.001 | 1.157 | 1.789 |  | |
| Q5 | 1.441 | 0.155 | 3.39 | 0.001 | 1.167 | 1.780 |  | |

Note: All analyses adjusted for age, sex, marital status, qualifications, total sleep time, sedative use.
